# Parasocial Interactions and Relationships in Early Adolescence

**DOI:** 10.3389/fpsyg.2017.00255

**Published:** 2017-02-23

**Authors:** Tracy R. Gleason, Sally A. Theran, Emily M. Newberg

**Affiliations:** Department of Psychology, Wellesley College, WellesleyMA, USA

**Keywords:** parasocial interactions, parasocial relationships, imagination, relationships, adolescence, gender

## Abstract

Parasocial interactions and relationships, one-sided connections imagined with celebrities and media figures, are common in adolescence and might play a role in adolescent identity formation and autonomy development. We asked 151 early adolescents (*M*_age_ = 14.8 years) to identify a famous individual of whom they are fond; we examined the type of celebrities chosen and why they admired them, and the relationships imagined with these figures across the entire sample and by gender. Adolescents emphasized highly salient media figures, such as actors, for parasocial attention. While different categories of celebrities were appreciated equally for their talent and personality, actors/singers were endorsed for their attractiveness more so than other celebrity types. Most adolescents (61.1%) thought of their favorite media figures as relationship partners, and those who did reported more parasocial involvement and emotional intensity than those who did not. Gender differences emerged in that boys chose more athletes than girls and were more likely to imagine celebrities as authority figures or mentors than friends. Celebrities afforded friendship for girls, who overwhelmingly focused on actresses. Hierarchical parasocial relationships may be linked to processes of identity formation as adolescents, particularly boys, imagine media figures as role models. In contrast, egalitarian parasocial relationships might be associated with autonomy development via an imagined affiliation with an attractive and admirable media figure.

## Introduction

Parasocial interactions and relationships (PSI/PSR) are symbolic, one-sided social ties that individuals imagine with media figures and celebrities (Horton and Wohl, 1956). Research on these parasocial processes has primarily focused on their explanatory power vis a vis individual differences in media use and consumption. While much of the research in this area has focused on undergraduate samples, and a growing body of work is examining these processes in children (e.g., Rosaen and Dibble, 2008; Bond and Calvert, 2014; Calvert et al., 2014; Brunick et al., 2016) the nature of these processes in adolescence is of particular interest for two reasons. First, adolescents demonstrate greater attention to and preoccupation with media figures and celebrities relative to other age groups (Giles, 2002; Giles and Maltby, 2004; Maltby et al., 2005), and adolescent PSI tends to be intense (Cohen, 2003; Klimmt et al., 2006). Second, theoretically, parasocial processes might play a role in helping adolescents address the tasks of this developmental period, such as identity formation and the development of autonomy from parents (Giles and Maltby, 2004). Combined with the fact that parasocial processes appear to follow similar patterns of formation and maintenance as real interactions and relationships (see, in particular, Rubin and McHugh, 1987; Schiappa et al., 2007), these findings suggest that the nature of adolescent parasocial processes might be of interest in their own right, not just in relation to media consumption but as a reflection of the social concerns of this developmental period. Consequently, the goal of the current study was to examine adolescent parasocial processes from a developmental relationships perspective.

Hartup’s (1995) work on friendship provides a framework for our approach. He identified three different “faces” of friendship: (1) involvement in friendship, (2) the identity of those friends, and (3) the qualities of the relationships; and demonstrated how each “face” related to different sets of important developmental outcomes. Theoretically, a similar approach could be applied to research on parasocial processes: examining the adolescent’s involvement in PSI/PSR, the identities of celebrities chosen for parasocial attention, and the qualities of the relationships imagined with media figures. To date, much of the extant research on parasocial processes has focused on relating degree of involvement in PSI/PSR to other variables, such as media consumption, loneliness, and attachment style (e.g., Rubin et al., 1985; Adams-Price and Greene, 1990; Greenwood and Long, 2011), which is akin to the first of Hartup’s three faces (i.e., involvement in friendship). Much less work has focused on either the identities of celebrities chosen for parasocial attention or the characteristics of the relationships imagined with them. In fact, to our knowledge, no work has asked the question of whether or in what ways adolescents imagine their favorite celebrities as relationship partners *per se*. These questions are of interest given that individual differences in adolescents’ choices of media figures and the sorts of relationships they imagine with them (if they do) might provide clues as to the psychological significance of the phenomenon and its relation to adolescent social development beyond those suggested by involvement in PSI/PSR alone.

### The Identities of Adolescents’ Celebrity Choices

The psychological significance (if any) of the specific media figures whom adolescents choose for PSI/PSR has received little attention (Turner, 1993; Giles and Maltby, 2004). While previous research has categorized individuals’ objects of PSI/PSR in terms of how realistic they are (Rosaen and Dibble, 2008; Tsay-Vogel and Schwartz, 2014) or examined variables relating to PSI within specific groups like newscasters (Rubin et al., 1985) and race car drivers (Hartmann et al., 2008), little attention has been given to choices of media figures according to vocational identities (e.g., athlete, actor). On the one hand, the choice of a popular actress versus a professional athlete might be incidental to the nature and function of parasocial processes in an adolescent’s life, or perhaps reflect preferences solely as a function of an adolescent’s developing gender identity. On the other hand, variation in celebrity choices and reasons for choosing particular celebrities might suggest developmental functions of PSI/PSR. Theoretically, as adolescents begin to construct their autonomous selves and engage in identity formation, parasocial processes might present identities for consideration and help individuals develop their own perspectives (Giles and Maltby, 2004; Madison and Porter, 2015)—meaning that media figure choices might be meaningful. For example, an adolescent girl in the throes of autonomy development might engage in PSR with an attractive actress, who affords an alternate and attractive affiliation to that provided by her parents (Adams-Price and Greene, 1990; Giles and Maltby, 2004; Klimmt et al., 2006). Alternatively, she might undertake PSR with a soccer star, who affords an imagined coach for her own achievement goals.

To date, only a handful of studies have examined the types of individuals chosen for parasocial attention and the rationale for these choices. Boon and Lomore (2001) categorized the vocations of specific media figures chosen for parasocial attention by young adult participants. These authors noted a high prevalence of actors (38.7%) and musicians (30.7%), the inclusion of deceased media figures (e.g., John Wayne), and infrequent mention of non-artistic celebrities such as Albert Einstein and Bill Gates. However, the authors did not explore the appeal of media figures in a chosen category or the types of relationships imagined.

A couple of studies suggest that choices of media figures for parasocial attention might be psychologically meaningful. For example, Cohen (1999) examined Israeli teenagers’ PSI/PSR with fictional characters on a popular television serial and the teenagers’ rationales for their choices. Adolescents preferred the teenage and young adult characters on the show over older characters and imagined young characters as friends. Adolescents appreciated their chosen characters’ attractiveness, personality, and to some extent, their social relationships and actions. Similarly, Turner (1993) studied variables contributing to PSI in undergraduates by asking them to report on soap opera characters, newscasters, or comedians. Participants who perceived attitude homophily with celebrities reported the greatest PSI regardless of type of media figure. However, other variables suggested that PSI was specific to celebrity type. High PSI in those reporting on newscasters was associated with participants’ perceptions of homophily in background with the stars, but for those reporting on soap opera characters, high PSI was associated with a lower inclination to communicate with real others. High PSI with comedians correlated with high positive self-evaluations.

In Turner’s (1993) and Cohen’s (1999) studies, participants were limited in their choices of celebrities either to a single show or to a specific category of celebrity, respectively. We expected that allowing adolescents to select their favorite media figures from any domain might elicit a wider range and variety of preferred celebrity types and reasons for liking them, perhaps highlighting individual differences in the psychological meaning of these choices.

As actors and musicians are salient media figures, we hypothesized that adolescents would endorse them at high rates, similar to those of the young adults in Boon and Lomore’s (2001) study, with low rates of less visible or non-artistic celebrities. However, we speculated that, in our sample, deceased media figures might not be represented, as currently popular celebrities would be modeling values and characteristics with contemporary appeal to adolescents beginning a phase of identity exploration. We also expected that media figures’ vocations would vary along with the reasons adolescents liked them; highly visible celebrities might be admired for external characteristics such as appearance, and media figures such as athletes or non-artists might be appreciated mostly for their talents. We were not sure whether adolescents would endorse internal characteristics (e.g., friendliness) as important.

### Parasocial Relationships in Adolescence

In addition to exploring the types of celebrities chosen for parasocial attention, we also investigated whether adolescents’ reports of parasocial processes might vary according to the distinction discussed by Schramm and Hartmann (2008) between PSI and PSR. Specifically, although many adolescents have favorite media figures and might imagine interactions with them during media consumption (PSI), most likely a smaller proportion engage in parasocial processes beyond the viewing experience, conceptualizing the media figure in relationship terms (PSR; Madison and Porter, 2016). If so, PSR might be differentiated, just as real relationships are, by how they are construed. After all, PSI/PSR emerges not just in relation to liked characters, but also in relation to those who participants feel neutral about (Tian and Hoffner, 2010) or even actively dislike (Dibble and Rosaen, 2011). Such qualitative variation is consistent with the fact that PSR has been tied theoretically to functions associated with real social networks, such as fulfilling social needs for shy individuals (Vorderer and Knobloch, 1996, as cited in Klimmt et al., 2006) or providing models for self-concept development in adolescence (Adams-Price and Greene, 1990).

The issue of variation in imagined relationships seems of particular relevance for an adolescent sample. In comparison to adults or even undergraduates, the age differences between young adolescents and their favorite stars are greater. Adults have described media figures as akin to neighbors (Gleich, 1996, as cited in Giles, 2002), associated with affiliative and egalitarian attachment needs (Cole and Leets, 1999; Cohen, 2004; Greenwood and Long, 2011). Adolescent relationships with celebrities, in addition to or instead of friendship, might afford supportive, hierarchical relationships, such as those adolescents often form with mentors, coaches, or other non-parental adults. Given the age differences between adolescents and most media figures, we expected adolescents to report more hierarchical than egalitarian PSR.

Despite our expectation that PSR in adolescence might often be construed as hierarchical, we hypothesized that any variation we did find might be systematic and psychologically meaningful. First, we expected that variations in PSR might correspond to the reason why adolescents liked celebrities. For example, we speculated that hierarchical PSR might be related to appreciation of a media figure’s talent (e.g., athleticism). In contrast, if a star was admired for his/her physical attractiveness, the resulting PSR might be in relation to affiliative needs and thus construed as egalitarian. Second, we hypothesized that adolescents who described their favorite celebrities in relationship terms would show greater parasocial involvement and emotional intensity than those who did not, and third, that these same group differences would emerge for parasocial activities (e.g., reading about the star online, discussing the star with friends).

### Gender

Given that male and female adolescents prefer different television characters (Cohen, 1999), we expected that boys’ and girls’ favorite celebrities would differ. Because of the current prominence of men’s versus women’s sports, we hypothesized that boys would be more likely than girls to identify athletes as objects of parasocial attention and that relatedly, girls would be more likely than boys to choose actresses. We also hypothesized gender differences in PSR. As females report higher frequency and intensity of engagement in parasocial activities than males (Hoffner, 1996; Cohen, 2003; Maltby et al., 2005), we expected higher rates of PSR and higher involvement and emotional intensity in girls than boys. We also derived hypotheses based on literature on mentoring relationships in adolescence since we postulated that adolescent PSR might be construed that way. According to Rhodes (2002, as cited in Darling et al., 2006) boys prefer mentors who participate in activities with them, while girls desire emotional closeness and connection in mentoring relationships. Consequently, we hypothesized that boys might appreciate talent in imagined mentors more than girls, and that girls’ intimacy-seeking might make them more likely than boys to engage in parasocial activities privately.

### Summary of Aims and Hypotheses

Our study had three major aims: (1) to examine adolescents’ choices of media figures and celebrities for parasocial attention, (2) to explore the prevalence and construal of PSR in an adolescent sample, and (3) to ascertain whether gender differences emerged in adolescent parasocial processes. With respect to adolescents’ choices of media figures and celebrities, we hypothesized that highly salient celebrities, such as actors and musicians, would be frequently mentioned, with lower rates of endorsement for non-artistic celebrities or deceased media figures. We also expected correspondence between type of celebrity chosen and the qualities adolescents associated with them, in that highly visible celebrities would be associated with appearance, whereas athletes and non-artists would be appreciated for their talents. For PSR, we expected adolescence who engaged in PSR to report more hierarchical than egalitarian relationships, but to the extent that variation appeared, we expected hierarchical PSR to be associated with appreciation of a celebrity’s talent and egalitarian PSR to be associated with appearance. We also expected PSR to be associated with high involvement, intensity, and investment in parasocial activities. As for gender differences, we expected greater endorsement of athletes among boys and actresses among girls, higher rates of involvement, intensity, and PSR in girls than boys, and for boys to appreciate their celebrities for their talents more often than girls. Lastly, we expected girls to be more private about their parasocial activities than boys.

## Materials and Methods

### Participants

The initial sample was 107 girls (*M*_age_ = 14.83, *SD* = 0.35) and 61 boys (*M*_age_ = 14.92, *SD* = 0.42) in ninth grade at a public high school in a US Northeastern suburb. However, seven girls (6.5%) and eight boys (13.1%) did not identify a celebrity of whom they were particularly fond, so the final sample included 100 girls (*M*_age_ = 14.83, *SD* = 0.35) and 53 boys (*M*_age_ = 14.88, *SD* = 0.38). This ratio reflects the gender ratios in much of the research on PSI (Cohen, 1999, 2003, 2004; Cole and Leets, 1999; Maltby et al., 2006; Derrick et al., 2008, 2009). Adolescents were 73% Caucasian, 12% Asian, 8% Biracial, 2% Latino, 2% African-American, and 1% Native-American (2% chose “Other” or did not respond), and approximately 75% had at least one parent who had graduated college. Participation (72.3%) was solicited through a letter and consent form sent to parents through the school. Participants received a $5 gift certificate for ice cream, and the school received a donation. This study was carried out with the approval of and in accordance with the recommendations of the Wellesley College Ethics Review Board with written informed consent from parents and assent from all participants in accordance with the Declaration of Helsinki.

### Measures

A survey addressed aspects of (PSI/PSR) including identification of celebrities chosen for parasocial attention and the range and variation in quasi-relationships imagined with these celebrities. We investigated whether celebrity vocation or relationships imagined were related to the characteristics adolescents admired in their chosen celebrities, as well as emotional and behavioral manifestations of PSI/PSR, such as participants’ level of involvement in parasocial activities, the emotional intensity of the experience, their dedication in finding out about their chosen celebrities, and whether they shared their interest in these media figures with friends and family. Participants were first asked to identify a same-sex celebrity of whom they were particularly fond (“Most teenage girls/boys have a favorite celebrity or a favorite character from TV, film, or pop culture: which FEMALE/MALE CELEBRITY are you particularly fond of?”). We focused on same-sex celebrities for consistency with prior research (e.g., Derrick et al., 2008).

#### Celebrity Type

Media figures named were categorized into five *celebrity types*: 1 = *actors*, 2 = *athletes*, 3 = *singers*, 4 = *general celebrities* (e.g., talk show host Oprah), and 5 = *writers*. Celebrities that fit into multiple categories (e.g., Oprah is also an actress) were coded according to the category for which they were best known. Two independent coders scored all responses; reliability was high (kappa = 0.92). Disagreements were resolved by discussion.

#### PSR Type

Adolescents were asked, “How do you like to think of this person [the celebrity chosen]? As a…” and then were asked to circle one of the following responses: sister or brother, best friend, parent, teacher, babysitter, or other. The “other” category was overwhelmingly completed with the following responses: friend, celebrity, and role model. Thus, we recoded sister or brother, best friend, and friend as indicative of an egalitarian pseudo-relationship, parent, teacher, babysitter, and role model as hierarchical pseudo-relationships, and “celebrity” as relating to the person as just a media figure, not as a relationship partner.

#### Characteristics

Participants were presented with a list of adjectives and asked to circle all those that represented qualities that they admired in their chosen celebrity. A small focus group, including two experts on adolescence and an undergraduate, generated these adjectives as words describing characteristics typically admired in celebrities. Adjectives included *funny, appearance, sense of style, talented, caring, charming, beautiful, thoughtful, friendly, generous, entertaining, kind, interesting, smart, charismatic, outgoing*, and *good-looking.* Participants were also given space to list their own adjectives. An exploratory principal axis factor analysis, with promax rotation (SPSS), revealed a three-factor solution that explained 37.14% of the variance. Six adjectives loaded on a factor we labeled *personality*: generous (0.76), kind (0.65), outgoing (0.42), caring (0.62), thoughtful (0.72), and friendly (0.52). A second factor, *appearance*, included beautiful (0.83), appearance (0.56), good-looking (0.78), and sense of style (0.52); and a third factor, *talent*, included talented (0.52), entertaining (0.55), interesting (0.54), and charismatic (0.44). Reliabilities for these factors were 0.79, 0.77, and 0.59, respectively. The adjectives *funny* and *smart* did not load onto any factor, and *charming* loaded weakly onto both personality and talented, so these three adjectives were not included in the analyses.

Twenty-five adolescents in the sample generated adjectives of their own, but no single adjective was mentioned frequently enough for further analysis. Twelve adjectives generated (48%) could have been categorized into one of our existing terms; we did not recode them as we did not want to misinterpret adolescents’ intentions in listing them under “other.” Eight adjectives referenced talent (e.g., “her voice,” “athletic”), three related to appearance (e.g., “bald and full bodied”), and one adolescent wrote “outgoing,” despite that adjective being on the printed list. Six generated characteristics were general (e.g., “awesome,” “cool”), and four referred to the celebrity’s inner strength or confidence (e.g., “confidence and pride,” “strong-willed,” “badass”). Two cited “realistic” as reasons for admiration, and one person wrote “motivated.”

#### Involvement

Participants completed a commonly used revision (Rubin and Perse, 1987; Auter, 1992; Cole and Leets, 1999) of the 20-item Parasocial Interaction Scale (Rubin et al., 1985) that applies to media figures generally rather than just newscasters like the original version. Items describe behaviors and attitudes toward a favorite media figure and are measured on a 5-point Likert scale (1 = *disagree strongly* to 5 = *strongly agree*). Scores were computed by averaging item responses. This scale has been demonstrated to have good construct validity and reliability (Auter, 1992; alpha for this study = 0.91). Although the scale specifically references PSI and emerged from research specific to parasocial processes in the context of media use, we refer to it as a measure of parasocial involvement because it includes questions that construe parasocial process in relationship terms (e.g., “I think this person is like an old friend”).

#### Emotional Intensity

We developed three items to measure emotional intensity of parasocial processes: (a) how strongly do you feel about him/her, (b) how connected do you feel to him/her, and (c) how well do you feel you know him/her? Items were measured on a 5-point Likert scale (1 = *not at all* to 5 = *very much*) and were averaged. An exploratory principal axis factor analysis, direct varimax rotation (SPSS), supported a one-factor solution, with factor loadings of 0.70, 0.88, and 0.67, respectively; this factor accounted for 56.92% of the variance and showed good reliability (Cronbach’s alpha = 0.79).

#### Dedication

We developed a 7-item scale concerning how dedicated adolescents were to thinking about and finding out about their favorite celebrity measured on a 4-point Likert scale (1 = *less than once a week*, 2 = *several times a week*, 3 = *once a day*, 4 = *several times each day*): (a) How often do you think about him/her? (b–e) what do you use to find out about this person’s real life? TV, Internet, Magazines, Friends; (f) How often do you imagine meeting this person? and (g) How often do you think about the kind of advice this person would give you if you went to her with a problem? An exploratory principal axis factor analysis, direct varimax rotation (SPSS), supported a one-factor solution, with factor loadings of 0.54, 0.69, 0.89, 0.39, 0.78, 0.53, and 0.51, respectively; this factor accounted for 40.56% of the variance and showed good reliability (Cronbach’s alpha = 0.81).

#### Sharing

Four items assessed whether adolescents’ shared their interest in the media figures they named with friends and family: “Do your friends/Does your family know that you like this person?” and “Do you and your friends/family talk about him/her together?” Responses were scored 1 = yes, 0 = no for each of the four questions and averaged. An exploratory principal axis factor analysis, direct varimax rotation (SPSS), supported a one-factor solution, with factor loadings of 0.43, 0.64, 0.51, and 0.61, respectively; this factor accounted for 30.52% of the variance and showed modest reliability (Cronbach’s alpha = 0.63).

### Procedure

Consent forms were distributed 2 weeks prior to data collection; participants completed assent forms. Surveys took approximately 45 min and were completed on paper during a 52-min class period. Researchers were present to supervise and answer questions.

## Results

Results are reported in three sections. First, we described the nature of adolescents’ parasocial activities, including the celebrities chosen and the characteristics adolescents endorsed as admirable in these media figures. Next, we described the types of relationships (if any) that adolescents reported imagining with their chosen media figures. Lastly, we explored the content of adolescents’ parasocial processes, including relations between PSR and admired characteristics, level of involvement in parasocial processes, emotional intensity of the experience, dedication to following media figures, and the extent to which adolescents shared these interests with others. All analyses were conducted within the context of gender.

### Celebrities Chosen for Parasocial Attention

Frequencies with which celebrity types were endorsed are displayed in **Table 1**. Our hypothesis that adolescents would endorse actors and singers at similar rates to young adults was not supported owing to the overwhelming tendency of adolescents to name actors. Media figures in other categories were infrequently named, as hypothesized. Boys, however, named athletes at similar rates to the adult sample from Boon and Lomore (2001), although their study elicited dancers and ours did not. Contrary to our expectation that adolescents would be solely focused on living stars, two adolescents (1.19%) named deceased media figures (Marilyn Monroe and Audrey Hepburn). Also, unlike the adult sample, whose “other” category included a photographer, entrepreneurs, a movie director, a physicist, an evangelist, a cancer research fundraiser, and Princess Diana (Boon and Lomore, 2001), every celebrity in our “general” category was either a talk show host or a comedian with one exception (Linus Torvalds, inventor of the Linux kernel). Lastly, as in the adult sample, few writers were named. Consequently, for the analyses that follow, we chose to include the writers in the category with “general” celebrities. Our rationale was that although writers are not performers like talk show hosts or comedians, they do provide entertainment without taking on other roles.

**Table 1:** Frequencies of endorsement of celebrity type and parasocial relationship quality.

Descriptive variables	Girls (*n* = 100)%	Boys (*n* = 53)%	Overall (*N* = 153)%	Young adults^a^ (*N* = 79)%
**Celebrity type**				
Actor	77.0	50.9	68.0	38.7
Singer/musician	17.0	18.9	17.6	30.7
Athlete	0.0	15.1	5.2	14.7b
Other (e.g., talk show host, comedian)	5.0	13.2	7.8	12.0
Writer	1.0	1.9	1.3	4.0
**Parasocial Relationship Quality**	(*n* = 95)	(*n* = 49)	(*n* = 144)	
Friend	31.6	20.4	27.8	
Authority figure	22.1	55.1	33.3	
Celebrity	46.3	24.5	38.9	


*^a^From Boon and Lomore (2001); includes 40 females and 39 males. ^b^Category includes dancers for this sample.*

We hypothesized that celebrity choices would differ by gender; this hypothesis was supported, χ^2^(3, *N* = 153) = 21.75, *p* < 0.001, Cramer’s *V* = 0.38. As expected, the gender difference was driven by boys’ more frequent endorsement of athletes (no girls named athletes as their favorite celebrities), girls’ overwhelming preference for actresses, and the tendency for boys to endorse celebrities in the “other” category somewhat more than girls (mostly comedians; see the top half of **Table 1**). For girls, the most commonly named media figures were Jennifer Aniston (*n* = 13), Jennifer Garner, Angelina Jolie, and Reese Witherspoon (*n* = 5 for each), and for boys, the most commonly named media figures were David Ortiz (*n* = 3), Tom Brady, Dave Chappelle, Johnny Depp, Ed Norton, and Kiefer Sutherland (*n* = 2 for each).

We next examined whether celebrity type related to the admired characteristics that adolescents associated with them. Our hypothesis that highly visible celebrities such as actors and singers would be admired for their appearance, and that athletes and non-artists would be endorsed for talent, was partially supported. We ran a MANOVA using celebrity type and gender as factors and the three characteristics (personality, appearance, and talent) as dependent variables. No main effects emerged for personality and talent, but endorsements of appearance differed significantly by celebrity type, *F*(3,146) = 5.47, *p* = 0.001, $\eta_{p}^{2}$ = 0.10. As predicted, *post hoc* pairwise comparisons (LSD) revealed that actors (*M* = 0.56, *SD* = 0.36) and singers (*M* = 0.50, *SD* = 0.40) did not differ from each other but were endorsed for appearance more than athletes (*M* = 0.03, *SD* = 0.09) or general celebrities (*M* = 0.16, *SD* = 0.23; all *p*s ≤ 0.002), who also did not differ. No celebrity type by gender interactions emerged for admired characteristics.

### Parasocial Relationships Imagined

Of the 153 adolescents who identified their favorite celebrities, 144 (94.12%) provided descriptions of how they thought of these media figures vis à vis themselves (see bottom of **Table 1**). Because of the age differences between adolescents and their favorite media figures, we hypothesized that a greater proportion of participants would conceptualize their favorite celebrities in hierarchical (i.e., authority figure) than egalitarian (i.e., friend) terms. This hypothesis was not supported; overall, the proportions of adolescents seeing their favorite celebrities in these ways or simply as celebrities did not differ significantly from a chance distribution χ^2^(2, *N* = 144) = 2.67, *p* = 0.264, Cohen’s *w* = 0.14. However, significant gender differences emerged, albeit not in line with our expectations (see bottom of **Table 1**). We hypothesized that girls would report higher rates of PSR (i.e., thinking of celebrities in relationship terms rather than just as celebrities) than boys, but instead, a higher proportion of boys (75.5%) than girls (53.7%) reported engaging in PSR. In addition, boys were more likely than girls to think of celebrities as authority figures, and when girls did imagine a relationship, they reported friendships more so than authority figures, χ^2^(2, *N* = 144) = 15.97, *p* < 0.001, Cramer’s *V* = 0.33.

### Content of Parasocial Processes and Relationships

To describe the content of adolescents’ parasocial processes, we began by examining average scores for the endorsement of admired characteristics (i.e., personality, appearance, and talent), parasocial involvement, emotional intensity, dedication, and sharing as well as correlations between these variables within gender (**Table 2**). Scores for the involvement and intensity variables fell in the lower half of the possible range, suggesting normative levels of engagement in parasocial activities. Adolescents reported a level of dedication to finding out about and/or thinking about their favorite celebrity that averaged between “less than once a week” and “several times a week” on the scale, and little sharing of their interest in the celebrity with either friends or family.

**Table 2:** Overall means (M) and Standard deviations (SD) and Correlations between measures of parasocial processes within gender.

Variable	*M*	*SD*	1	2	3	4	5	6	7
1. Personality	0.34	0.33	–	0.28*	0.34*	0.32*	0.32*	0.20	0.06
2. Appearance	0.48	0.38	0.35***	–	0.44***	0.02	0.10	0.34*	-0.09
3. Talent	0.57	0.32	0.24*	0.28**	–	0.25+	0.17	0.03	0.11
4. Involvement	2.69	0.75	0.12	0.10	-0.01	–	0.63***	0.16	0.44**
5. Intensity	2.05	0.91	0.21*	0.09	-0.09	0.63***	–	0.26+	0.28+
6. Dedication	1.37	0.54	-0.02	0.10	-0.13	0.18+	0.28**	–	0.06
7. Sharing	0.51	0.31	0.06	-0.03	0.06	0.23*	0.20*	0.12	–


*Correlations for boys are above the diagonal, and correlations for girls are below the diagonal. +*p* ≤ 0.10. *^∗^p* ≤ 0.05. ^∗∗^*p* ≤ 0.01. ^∗∗∗^*p* ≤ 0.001.*

For both genders, involvement and intensity were correlated with each other, as were celebrities’ admired characteristics. Endorsement of personality as an admired characteristic was correlated with intensity, whereas involvement was positively correlated with sharing. Other correlations were unique within gender. For girls, significant positive correlations emerged among intensity and dedication and sharing (but not between the latter two). Girls’ reports of involvement correlated marginally with dedication. Boys’ endorsement of appearance as an admired characteristic correlated positively with dedication and personality correlated positively with involvement. Marginal positive correlations also emerged for boys between talent and involvement, and among intensity and dedication and sharing (but again, not between the latter two).

To examine how admired characteristics, involvement, intensity, dedication, and sharing related to relationship types imagined with celebrities, we conducted a set of two-way MANOVAs using relationship type and gender as factors. The first MANOVA used the admired characteristics as dependent variables, the second used involvement and emotional intensity, and the third used dedication and sharing. We used this approach rather than conducting a single analysis predicting all of the dependent variables at once because of sporadic missing data that reduced our sample size in a single MANOVA. Also, although unconventional, we present the main effects of PSR type for all the parasocial processes first, followed by the main effects of gender, and then present the single interaction that emerged. With so little interaction between our independent variables, this presentation best illustrates the patterns of engagement in parasocial processes as a function of PSR type and of gender.

#### PSR Type

We hypothesized that hierarchical PSR might be more related to talent than egalitarian or no PSR, but no main effects of PSR type emerged for the MANOVA using the three admired characteristics (personality, appearance, and talent) as dependent variables (**Table 3**). However, as expected, significant main effects emerged for both parasocial involvement and emotional intensity in relation to PSR type. *Post hoc* tests (LSD) revealed that for both involvement and intensity, means for media figures seen as celebrities were significantly lower than those for media figures imagined as friends or authorities (all *p*s ≤ 0.001); the latter two groups did not differ (see **Table 3** for means). Our hypothesis that PSR type would be associated with parasocial activities was partially supported for dedication, not for sharing. *Post hoc* analyses showed that means for the no PSR group were significantly lower than those for parasocial friends (*p* = 0.022) and marginally lower than those for parasocial authorities (*p* = 0.088); friends and authorities did not differ (*p* = 0.476; see **Table 3** for means).

**Table 3:** Means (and standard deviations) and main effects of parasocial relationship type for parasocial processes.

	PSR type					
				
Parasocial processes	Friend	Authority	Celebrity	*F* (2,138)^a^	*p*^b^	$\eta_{p}^{2}$
Personality	0.39 (0.34)	0.33 (0.29)	0.28 (0.33)	2.07	0.131	0.03
Appearance	0.59 (0.38)	0.36 (0.36)	0.50 (0.40)	1.81	0.168	0.03
Talent	0.53 (0.32)	0.57 (0.34)	0.59 (0.30)	0.30	0.742	0.00
Involvement	2.93a (0.76)	2.87a (0.61)	2.36b (0.73)	12.34	0.000	0.15
Intensity	2.28a (0.85)	2.23a (0.94)	1.65b (0.71)	9.92	0.000	0.13
Dedication	1.48a (0.63)	1.41a,b (0.53)	1.23b (0.40)	4.12	0.018	0.06
Sharing	0.49 (0.33)	0.57 (0.30)	0.45 (0.31)	1.29	0.280	0.02


*Within parasocial processes, values with different superscripts differ significantly (*p* ≤ 0.022). PSR, Parasocial relationship. ^a^Error *df* was 136 for involvement and intensity and 130 for dedication and sharing. ^b^*n* = 144 for personality, appearance and talent; 142 for involvement and intensity; and 136 for dedication and sharing.*

#### Gender

Main effects of gender emerged for personality and for appearance in that girls endorsed these factors more strongly than boys (*p*s < 0.001) but not for talent (see **Table 4** for means). Contrary to expectation, main effects of gender did not emerge for involvement, emotional intensity, or sharing; boys reported higher dedication than girls (*p* = 0.015; **Table 4**).

**Table 4:** Means (and standard deviations) and main effects of gender for parasocial processes.

	Girls			Boys					
		
Parasocial processes	*n*	*M*	*SD*	*n*	*M*	*SD*	*F* (1,138)^a^	*p*^b^	$\eta_{p}^{2}$
Personality	95	0.38	0.32	49	0.22	0.30	12.61	0.001	0.08
Appearance	95	0.61	0.36	49	0.23	0.31	32.09	0.000	0.19
Talent	95	0.58	0.30	49	0.56	0.36	0.19	0.663	0.00
Involvement	95	2.69	0.77	47	2.69	0.71	1.16	0.285	0.01
Intensity	95	1.93	0.80	47	2.21	0.99	1.39	0.240	0.01
Dedication	90	1.27	0.46	46	1.53	0.60	6.14	0.015	0.05
Sharing	90	0.46	0.29	46	0.59	0.35	3.71	0.056	0.03


*^a^Error *df* was 136 for involvement and intensity and 130 for dedication and sharing. ^b^*n* = 144 for personality, appearance and talent; 142 for involvement and intensity; and 136 for dedication and sharing.*

#### Interaction

Only one interaction emerged between PSR type and gender in any of the MANOVAs, in relation to talent, *F*(2,138) = 4.64, *p* = 0.011, $\eta_{p}^{2}$ = 0.06, but not quite as we had hypothesized. We expected that boys would appreciate talent in imagined mentors more than girls. Indeed, pairwise comparisons revealed that among boys, authority figures were valued for their talents more so than media figures seen as celebrities (*p* = 0.026), but friends did not differ from either group. However, the interaction was driven by the fact that boys’ ratings of talent were significantly lower than girls for media figures thought of as celebrities (*p* = 0.007), not by high ratings for authorities (see **Table 5** for means). Girls’ endorsement of talent did not differ between media figures considered as friends, authorities, and celebrities.

**Table 5:** Means (and standard deviations) for talent as a function of PSR type and gender.

	PSR type		
	
Gender	Friend	Authority	Celebrity
Girls	0.51 (0.32)	0.51 (0.32)	0.65^y^ (0.26)
Boys	0.60^ab^ (0.34)	0.62^a^ (0.36)	0.38^bz^ (0.36)


*Values with different superscripts a and b differ significantly *within* gender (*p* = 0.026); values with different superscripts y and z differ significantly *between* genders (*p* = 0.007).*

## Discussion

The findings presented here illustrate a nuanced picture of parasocial processes in adolescence. Specifically, the illustration of qualitative individual differences in (PSI/PSR) points out the systematically varied roles of imaginative processes in adolescent development. For example, the gender differences that emerged in adolescent parasocial processes might reflect variations in boys’ and girls’ social developmental priorities. Our results also suggest avenues for future research related to the kinds of relationships imagined with celebrities and what role they might play in adolescent development.

### Normative Parasocial Processes in Adolescence

Most of the adolescents invited to participate in this study chose a favorite celebrity, and responses to our measures of involvement and emotional intensity in parasocial processes fell into a moderate range. Adolescents reported thinking about and seeking information related to their favorite media figures maybe once a week or so, and few discussed these celebrities with real others. These results suggest that we accessed a normative form of this imaginative behavior that is consistent with a form of celebrity interest previously deemed developmentally appropriate for adolescents and unassociated with psychopathology (Adams-Price and Greene, 1990; McCutcheon et al., 2004; Maltby et al., 2006).

The celebrity types that adolescents chose differed somewhat from those chosen in research with undergraduates (Boon and Lomore, 2001). The fact that most adolescents focused on actors, with singers a close second, might simply reflect the vast media attention given to television/film stars. This attention, which focuses largely on actors’ wealth and glamor, might make actors’ public personae particularly attractive to adolescents involved in identity exploration. Similarly, the narrower range of general celebrities chosen by adolescents in comparison to the young adults of Boon and Lomore’s (2001) work might reflect the lower prominence of these individuals in popular media, or perhaps adolescents’ lower exposure to general celebrities relative to that of young adults. Regardless, the results indicate that despite the ease with which adolescents could seek exposure to or information on media figures through the Internet, many teens prefer stars they see enacting roles on film or television.

The gender differences in celebrity types chosen by adolescents clearly suggest a greater focus on athletes among boys than girls. The fact that not a single girl named an athlete is perhaps unsurprising given the lower media coverage of women’s versus men’s athletics. However, this discrepancy is worth further investigation given the connections that have emerged between parasocial processes and negative body image in adolescent and young adult women (Maltby et al., 2005; Greenwood, 2009). Greater salience of female athletes in the media could theoretically increase adolescent girls’ parasocial attention to female celebrities exemplifying healthy body images and behaviors that correspond to physical health. Such increases might be advantageous to young women’s development, given that individuals report making efforts to be more like the media figures with whom they engage in PSR (Sood and Rogers, 2000; Klimmt et al., 2006; Tian and Hoffner, 2010).

Aside from the differences owing to gender, the results relating to adolescents’ endorsement of various admired characteristics provide some clues as to why certain media figures might be preferred by adolescents. For example, endorsements of talent were highest, on average, of the three categories of characteristics. Admiration of a celebrity’s talent thus may be central to adolescent’s liking of a particular celebrity, a finding that is consistent with previous research highlighting the tendency of individuals to engage in parasocial activities with media figures who possess qualities they admire (Klimmt et al., 2006). In contrast, adolescent endorsements of appearance-related characteristics varied between the high salience media figures, actors and singers, on the one hand and the athletic/general media figures on the other. As most adolescents chose actors and singers, a focus on physical appearance (in addition to admiration of talent) might be considered typical in adolescence. To the extent that the emphasis on appearance is unassociated with athletes and general celebrities, an empirical question is whether the higher rates at which young adults chose media figures in these categories (Boon and Lomore, 2001) might signal a small developmental shift away from appearance as a priority for parasocial activities over the course of adolescence into young adulthood.

While actors and singers were appreciated for their attractive appearances more so than other media figures, in general, adolescents’ involvement (for boys) and emotional intensity (for both genders) in parasocial processes was related to admiration of celebrity personality characteristics, not attractiveness or talent. These correlations corroborate previous work finding a greater association between parasocial interaction and social rather than physical attraction (Rubin and McHugh, 1987), as well as research showing that understanding the attitudes and behavior of a media figure (i.e., his or her personality) is associated with investment in parasocial processes (Rubin and Rubin, 2001). These results also support the idea that (PSI/PSR) evolve in some ways that are parallel to real interactions and relationships (Rubin and McHugh, 1987; Perse and Rubin, 1989). For instance, relationship researchers emphasize the central role of attractiveness in social relationships, but theories of close relationship development also highlight the critical importance of reciprocity and information exchange, particularly with respect to a person’s attitudes, values, and feelings (Berscheid and Regan, 2005). If an adolescent imagines she is getting to know and to like a media figure’s personality, she may simultaneously experience increasing emotional investment in PSR. In contrast, celebrities’ talents and attractiveness did not correspond to parasocial intensity, meaning that these characteristics might be admired but less associated with emotion.

### Parasocial Relationships

The majority of adolescents (57.6%) reported thinking of their chosen celebrities in relationship terms. Those who did, regardless of the relationship type imagined, scored higher on measures of parasocial involvement and emotional intensity than the participants who thought of their favorite celebrities merely as such. What is more, as we hypothesized, adolescents who created egalitarian (and to a marginal extent, hierarchical) relationships with their favorite media figures also reported more dedication than did those adolescents whose favorite celebrities were seen as such. These findings are consistent with the conceptual distinction between PSI and PSR (Schramm and Hartmann, 2008), in that adolescents who engaged in PSR seemed to spend more time thinking about and investing emotional energy into these imagined relationships—specifically, outside the time spent in media use—than adolescents who did not consider their favorite celebrities in relation to themselves.

One purpose for these PSR presented in the literature is that they might play a role in identity formation (Adams-Price and Greene, 1990; Giles and Maltby, 2004). This idea is consistent with Erikson’s (1968) theory of so-called “secondary attachments,” in which adolescents imagine relationships and associate emotions with distant others. These relationships purposefully do not include reciprocity and are described as providing a safe forum for the adolescent to experiment with different ways of being. This interpretation is consistent with the correlations we found between the emotional intensity of parasocial processes and adolescents’ endorsement of characteristics related to personality.

Among those adolescents who conceptualized their favorite media figures in relationship terms, egalitarian and hierarchical relationships were reported with similar frequency (although a significant gender difference emerged; see below). These relationship types were not differentially associated with admired characteristics or the extent to which they were discussed with friends and family. Nevertheless, future research should attend to individual differences in the types of relationships imagined with media figures so as to establish whether these variations hold psychological or developmental significance.

### Gender Differences

Boys and girls did not differ in the extent to which they reported involvement or emotional intensity in parasocial processes, nor did they differ on the extent to which they discussed their favorite celebrities with friends and family. These findings run contrary to previous research that has suggested that parasocial processes are more intense among women than men, although much of this work emerged from undergraduate samples (e.g., Cohen, 2003; Maltby et al., 2005) rather than early adolescents. We also did not find higher rates of PSR in girls than boys as we had expected; in fact, a higher proportion of boys (75.5%) than girls (53.7%) thought of their favorite celebrities in relationship terms. Replication will be needed to establish whether this gender difference is characteristic of young adolescent samples.

Gender differences in the categories of favorite celebrities chosen and in the types of relationships that boys and girls described with them raise interesting questions regarding gender differences in the functions of parasocial processes in adolescence. In some ways, boys’ tendency to construe their PSR as hierarchical makes sense, as their celebrity choices tended to be men who were significantly older than the boys themselves. The fact that many boys saw these highly successful media figures as authority figures—perhaps as role models to emulate—again is consistent with Erikson’s assertion that PSR can be part of the developmental process of identity formation (Erikson, 1968). Earlier work (Adams-Price and Greene, 1990) has also suggested that adolescent males see their parasocial relationship partners as more agentic than themselves. Theoretically, the appeal of these particular media figures might be related to their success in their chosen fields. The tendency of boys who saw their favorite celebrities as authorities to endorse characteristics related to talent more so than boys who saw their favorite celebrities as such lends modest support to this interpretation.

Girls also chose celebrities significantly older than themselves, but they endorsed these relationships as hierarchical less frequently than boys. Instead, girls often imagined egalitarian relationships with their favorite media figures, but many girls did not report seeing them in relationship terms at all. Given that imagined intimacy with a same sex media figure has been positively correlated with reports of intimacy with a real friend (Greenwood and Long, 2011), when girls create egalitarian PSR, it might be indicative of autonomy development—specifically, the adolescent shift in social focus from parents to peers (Giles and Maltby, 2004). As girls draw more upon the peer context and develop intimacy in friendships in autonomy development, egalitarian PSR with a favorite celebrity might provide a corresponding imagined forum for simulating autonomy. Indeed, theoretically, choosing a talented, attractive, media personality for imagined affiliation might be ideal. Such a person projects the wisdom and self-assuredness of her actual age (which is probably not far from that of the adolescent’s parents), but the imaginary nature of the relationship means that it can be construed as egalitarian. Rhodes et al. (2006) suggested a similar concept for how mentors may shape identity formation in adolescents; they hypothesized that mentors give their mentees a framework for who they might become.

Finally, the gender differences with respect to dedication, and marginally to sharing, suggest that boys access celebrity-related media and discuss media figures with others more so than girls. These differences might have been driven in part by the greater proportion of boys than girls who imagined their favorite celebrities as relationship partners, and/or by the fact that boys also generally use media more than girls (Wartberg et al., 2015).

### Limitations

This study has several limitations. First, the smaller number of boys versus girls in the sample restricted the number and type of analyses that could be conducted. Second, we did not measure media use. Therefore, we cannot account for the extent to which media exposure either influenced the results (Schiappa et al., 2007; Singer and Singer, 2009). We also cannot assess the extent to which different types of media use (e.g., social media, television) might have influenced adolescents’ exposure to particular media figures and consequent choices. Third, we restricted adolescents to same-gender celebrities; some might have had a strong preference for an opposite-sex celebrity. Fourth, in retrospect, some of our admired characteristics, such as beautiful and caring, have gender-stereotyped connotations that affected their endorsement by boys.

### Conclusion

While imagined social relationships are unlikely to supersede real ones in importance, they might play a significant role in social development. Indeed, the variations in PSR both between and within genders support the notion that adolescents are imagining the relationships they need, whether egalitarian or hierarchical, and possibly in relation to gender differences in developmental goals. While research has emphasized important relations between parasocial processes and a wide array of psychological variables, the findings presented here emphasize that parasocial processes vary, both according to the celebrities chosen and the relationships imagined. At least in early adolescence, closer attention to this variation could provide clues as to the functional significance of this phenomenon in development, both in terms of understanding why these unilateral social ties are appealing as well as the meaning they might have at different developmental stages or in relation to the tasks of social development.

## Author Contributions

Data presented here were originally collected for an undergraduate honors thesis completed by EN under the direction of ST. EN and ST substantially contributed to the conception of the work and the acquisition and coding of the data. All authors contributed to the literature review. ST and TG substantially contributed to the conception of the study and to the interpretation and analysis of the data, the drafting of the study and revisions of the work.

## Conflict of Interest Statement

The authors declare that the research was conducted in the absence of any commercial or financial relationships that could be construed as a potential conflict of interest.
